# *Betula alba* Bark Extract and *Empetrum nigrum* Fruit Juice, a Natural Alternative to Niacinamide for Skin Barrier Benefits

**DOI:** 10.3390/ijms232012507

**Published:** 2022-10-19

**Authors:** Sandra Smiljanic, Cyril Messaraa, Virginie Lafon-Kolb, Nina Hrapovic, Nahid Amini, Christina Osterlund, Lene Visdal-Johnsen

**Affiliations:** 1 Oriflame Cosmetics, Swedish Research & Innovation Lab, Fleminggatan 14, 112 26 Stockholm, Sweden; 2 Oriflame Cosmetics, R&D Ltd., Bray Business Park, Kilruddery, A98 Y6W0 Bray, Ireland

**Keywords:** skin barrier, hydration, Scandinavian endemic plants, natural ingredient, *Betula alba*, *Empetrum nigrum*

## Abstract

The Scandinavian region is home to a unique biome with endemic plant species. The aim of this study was to explore this natural diversity and identify plant extracts providing positive skin barrier effects. Six plant extracts were identified as starting material. Following biochemical screening, two candidates outperformed the rest: *Betula alba* (BA) and *Empetrum nigrum* (EN). Quantitative PCR analysis showed that BA and EN upregulated barrier genes, when used individually and in combination. *Betula alba* increased AQP3 and OCLN protein expression, something niacinamide was incapable of. Additionally, the skin barrier was strengthened, evidenced by inhibition of KLK5 and hyaluronidase and showed strong antioxidant and anti-inflammatory activity through DPPH and COX2 inhibition, respectively. A first split-face clinical study was conducted using the combination of extracts versus placebo. There was a significantly better skin restructuring effect and corneocyte cohesion on the side treated with combined extracts. A second split-face clinical study assessed the combined extracts versus 3% niacinamide. Significant variations in skin hydration and TEWL were observed in favor of the extract treated side. In conclusion, we identified a natural alternative to niacinamide for improving skin barrier health, in Scandinavian plant extracts, which yield strong performance, but at a lower concentration.

## 1. Introduction

The skin consists of three layers, the epidermis, the dermis and the hypodermis, with each playing a role in protecting the body. The whole epidermis supports the skin barrier, although it is the outermost layer called stratum corneum (SC) that provide most of the skin barrier’s function [1]. The SC consists of corneocytes that are tightly held together by the lipid layer consisting of cholesterol, ceramides, and fatty acids. It sustains normal desquamation and skin elasticity by maintaining the skin’s water balance and attenuating external stresses [2]. Additionally, this layer contains keratin and natural moisturizing factors (NMFs). The skin acts as a physical and chemical barrier against external factors such as pathogens, toxins, pollution, ultraviolet rays, and dehydration. A healthy barrier maintains the integrity of the skin [1,2]. In fact, it is essential for good skin health, which is why it has been and still is the target for cosmetic skin care products.

A damaged skin barrier leads to dry, itchy skin and plays a role in skin conditions like atopic dermatitis and eczema [3]. To maintain healthy skin, adequate skin hydration is required. Trans-epidermal water loss (TEWL) is the amount of water that passively evaporates from the surface of skin. In healthy skin, TEWL is directly proportional to skin hydration. It can be affected by environmental (temperature, humidity, and ventilation) as well as intrinsic factors (cell senescence and telomere shortening), which is why these measurements must be performed under controlled conditions [4].

Tight junctions (TJs) are intramembrane multiprotein complexes that connect adjacent cells in endothelial and epithelial cells. Together with adherens junctions, desmosomes and gap junctions, TJs provide a permeability gradient to water, ions and other molecules as well as structural integrity to tissues. Tight junction structures are found in the stratum granulosum (SG), while the TJ proteins can be found in all skin layers. Together they maintain normal barrier function by preventing elevated TEWL in healthy skin. One of these TJ proteins is occludin (OCLN), which is primarily expressed in the SG, and is crucial for TJ stability and skin barrier function [3].

Aquaporins (AQPs) are other important proteins for skin barrier integrity. They act as channels to transport water into cells. Aquaporin 3 (AQP3) is expressed in the stratum basale and stratum spinosum and regulates epidermal structure and function [5]. It belongs to a subgroup of aquaporins called aquaglyceroporins, which transports not only water but also small molecules such as glycerol, thereby playing an important role in skin hydration [5,6].

Among skin care ingredients, niacinamide is well-established as it can be found in 15% of facial skin care products launched, a figure rising to 22% for the anti-ageing segment [7]. This is hardly surprising as niacinamide has a long history of use in skin and is hailed as a performing ingredient for skin barrier benefits [8]. It has been found to upregulate ceramide, sphingolipid, free fatty acid, and cholesterol synthesis in human keratinocytes. Its efficacy has been translated in vivo with a reduction of dry skin appearance, TEWL [9] and increased keratinocyte differentiation [10]. More recently, niacinamide has been shown to mitigate the release of pro-inflammatory lipids, such as arachidonic acid and prostaglandin D2, during skin barrier disruption [11].

Over the last decade, natural ingredients in skin care have become a key criterion for purchase by consumers [12], which has driven demand for the cosmetic industry to produce more natural products for topical application [13]. However, studies comparing well-known ingredients and natural alternatives are scarce. The aim of this study was to screen Swedish plant extracts and compare their effectiveness to so-called “gold standard” active ingredients, i.e., those with a long history of use and well established, proven efficacy, such as niacinamide. Various enzymatic assays were performed to assess the activity of the extracts, followed by in vitro testing. Finally, the efficacy of the combined extracts was supported by double-blind in vivo studies.

## 2. Results

### 2.1. The Extracts

To find a new natural product for topical application with skin barrier benefits, six plants were chosen, as seen in Table 1, to undergo a series of testing. These six plants are native Scandinavian flora and have historically been used in traditional medicine [14,15,16,17,18,19].

### 2.2. Biochemical Screening Results

The six chosen plant species are known to have antioxidant and anti-inflammatory activity [14,15,16,17,18,19]. The study therefore started by screening for kallikrein 5 (KLK5) inhibition, followed by hyaluronidase inhibition. Additionally, cytotoxicity was assessed before commencing with additional tests to ensure that the extracts were not cytotoxic.

**Figure 1:**
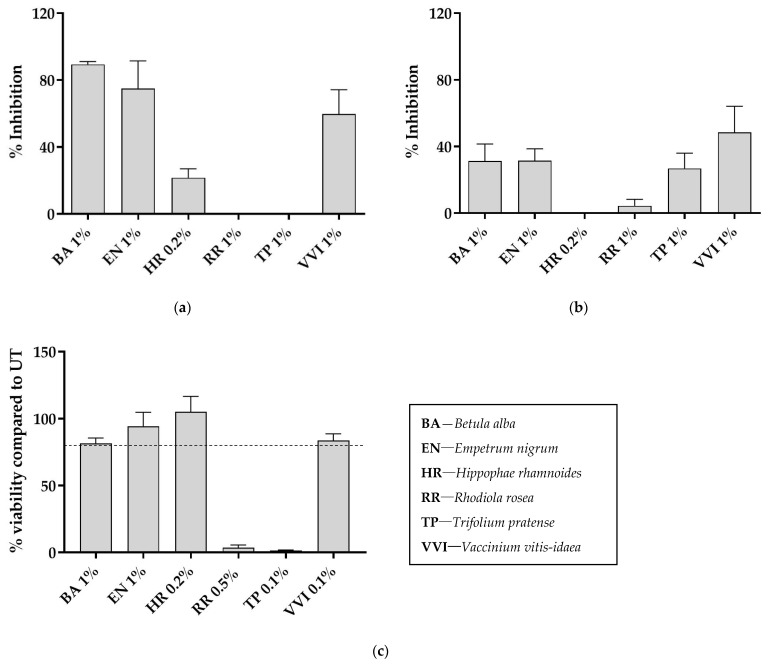
Screening results for the six extracts in (**a**) KLK5 inhibition assay (*n* = 2); (**b**) hyaluronidase inhibition assay (*n* = 2); and (**c**) viability assay (*n* = 3), where the dotted line represents 80% viability, which was the qualified test requirement for cytotoxicity. Values are displayed as mean (±SD).

Kallikrein 5 is a serum protease, important for skin barrier function. Excessive levels of KLK5 could result in inflammatory conditions such as atopic dermatitis and rosacea [20,21]. One percent of BA and EN inhibited 89% ± 2% (mean ± SD) and 75% ± 17%, respectively, of the KLK5 enzyme, VVI 60% ± 15% (at 1%), HR 22% ± 5% (at 0.2%) while RR and TP were not active, Figure 1a.

Hyaluronidase is an enzyme that breaks down hyaluronic acid. Less hyaluronic acid leads to dehydrated skin and weakens the skin barrier [22]. The results from the screen showed that the strongest hyaluronidase inhibition was exhibited by VVI (49% ± 16%), with moderate inhibition by EN (31% ± 7%), BA (31% ± 10%) and TP (27% ± 9%) at 1% extract, Figure 1b.

The enzymatic assay screening was followed by viability testing to evaluate the cytotoxicity of the six extracts. The qualified test requirement was reached if cell viability was ≥80%. Anything below 80% was considered cytotoxic. With this method, all extracts showed good viability except for RR and TP, see Figure 1c.

Based on this initial screening, HR, RR and TP extracts were excluded from further testing due to lack of activity and low viability. Additionally, VVI extract had solubility issues and was excluded for this reason.

### 2.3. In Vitro Testing

#### 2.3.1. Skin Barrier Gene Expression Analysis

With the two remaining extracts, the focus turned to skin barrier health and gene expression analysis for ten barrier genes. These genes all code for proteins known to be important for skin barrier function, see Table 2.

**Figure 2:**
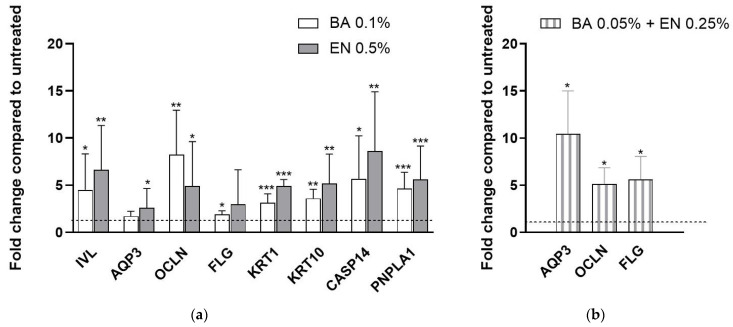
Gene expression analysis of skin barrier genes. Human primary keratinocytes were treated for 24 h with (**a**) single extracts: BA 0.1% (white) or EN 0.5% (grey) and (**b**) combination of extracts: BA 0.05% + EN 0.25% (striped). Relative gene expression was determined using the 2^−ΔΔCt^ method. Results are presented as fold change compared to untreated where (**a**) *n* = 5 and (**b**) *n* = 3 number of donors. Statistics was calculated on ΔCt values with (**a**) repeated measures ANOVA and post-test Tukey or (**b**) paired Student’s t-test where *p* * ≤ 0.05, *p* ** ≤ 0.01 and *p* *** ≤ 0.001. Fold change for untreated was set to 1. Values are displayed as mean (±SD).

It was found that 24 h treatment of human primary keratinocytes with 0.1% BA or 0.5% EN (solute concentration), significantly upregulated seven out of ten genes compared to untreated, see Figure 2a. When combining the extracts at 0.05% BA + 0.25% EN (solute concentration), it resulted in similar or even higher upregulation for three of the genes; *AQP3*, *OCLN* and *FLG*, compared to treatment with single actives, see Figure 2b.

#### 2.3.2. Skin Barrier Protein Expression

**Figure 3:**
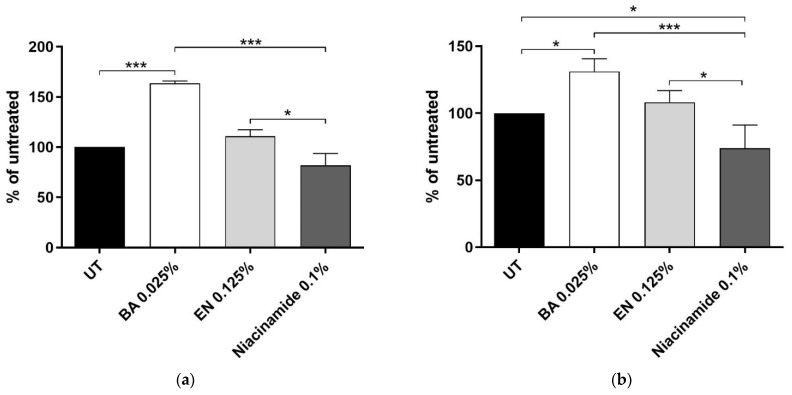
Protein expression of (**a**) AQP3 and (**b**) OCLN in human primary keratinocytes after 48 h treatment with BA or EN. As a reference, 0.1% niacinamide was used. Statistics was performed on normalized relative fluorescence intensity with repeated measures ANOVA and post-test Tukey where *p* * ≤ 0.05, and *p* *** ≤ 0.001. Untreated was set to 100% and *n* = 3 where *n* is the number of donors. Values are displayed as mean (±SD).

Next, it was examined if any of the barrier genes were translated into protein. After 48 h treatment with the actives, 0.025% solute concentration of BA resulted in higher levels of both AQP3 (Figure 3a) and OCLN (Figure 3b) protein compared to untreated (UT). Additionally, both extracts, BA and EN, resulted in significantly higher protein expression as compared to niacinamide. Representative density plot and histograms can be found in Figure S1.

### 2.4. Composition of the Extracts

The enzymatic and in vitro screening performed, showed that BA and EN had the strongest activity in these assays. For the two chosen extracts, the next step was to identify the compounds present in each of them.

Accurate mass measurement is a useful tool in the identification of phytochemicals in plant extracts, especially where a limited number of reference compounds are available [23]. In this study, BA and EN were analysed by UHPLC/Q-ToF and several compounds were tentatively identified based on accurate mass measurement and previous publications [15,24,25,26] and are listed in Table 3. Chromatograms can be found in Figure S2.

The presence of these compounds, i.e., phenolics and anthocyanins, indicate strong antioxidant and anti-inflammatory properties of the extracts [26,27] reaffirming the initial results seen in Figure 1.

### 2.5. Antioxidant and Anti-Inflammatory Effect

**Figure 4:**
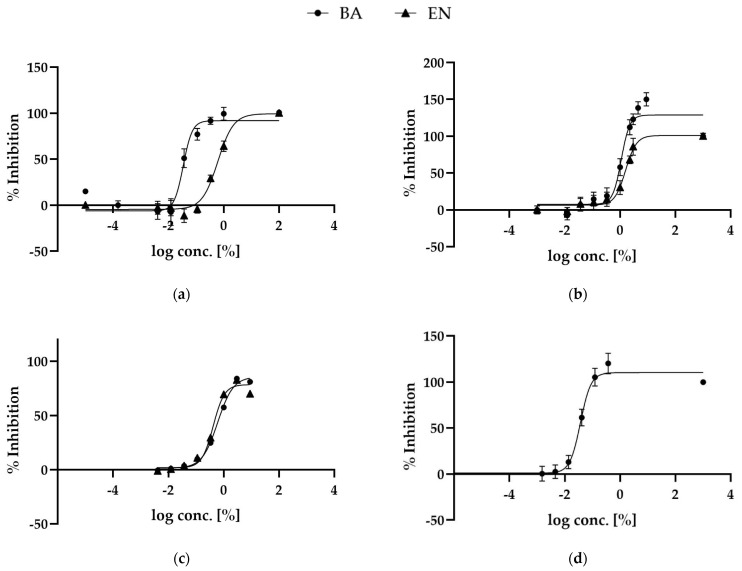
Summary of IC50 of the two extracts BA (circle) and EN (triangle) in (**a**) KLK5 (*n* = 3), (**b**) hyaluronidase (*n* = 3), (**c**) DPPH (*n* = 3) and (**d**) COX2 (*n* = 4) inhibition assays. Plotted values are displayed as mean (±SD).

The extracts were further investigated, looking at IC50 values for hyaluronidase and KLK5 inhibition assays. The hyaluronidase IC50 was determined for BA and EN to be 1.1% ± 0.2% (mean ± SD) and 1.6% ± 0.3%, respectively, Figure 4a. The IC50 for KLK5 inhibition assay was 0.03% ± 0.01% for BA and 0.6% ± 0.1% for EN, Figure 4b.

The antioxidant and anti-inflammatory activity of these two extracts has been extensively studied in the literature [14,15]. The extracts antioxidant activity was evaluated by measuring their capacity to scavenge the radical 2,2-Diphenyl-1-picrylhydrazyl (DPPH). The IC50 was determined to be 0.6% ± 0.1% for BA and 0.5% ± 0.1% for EN, Figure 4c. The anti-inflammatory activity of the two extracts was examined by COX-2 inhibition assay. Only BA showed strong inhibition of the COX2 enzyme with an IC50 of 0.04% ± 0.01%, Figure 4d. *Empetrum nigrum* did not show any activity.

These results demonstrate that both BA and EN extracts have good antioxidant and anti-inflammatory activities at low concentrations, with BA outperforming in enzymatic screening IC50 values.

### 2.6. Clinical Testing

#### 2.6.1. *Betula alba* and *Empetrum nigrum* Extracts versus Vehicle

**Figure 5:**
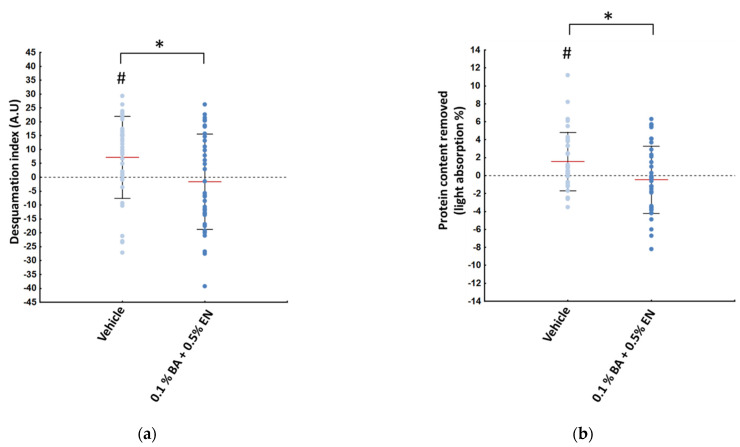
Desquamation index (**a**) and protein content removed from D-squames (**b**) after 28 days of treatment for the vehicle (light blue) and the BA + EN (dark blue) treated sides. * denotes a significant difference between the two treated sides, while # denotes a significant variation versus baseline measurements (paired Student’s *t*-test, *p* * ≤ 0.05). Red bars represent mean ± SD in black (*n* = 35).

Following 28 days of treatment with 0.1% BA + 0.5% EN extracts (solute concentration) on one side of the face and the vehicle alone on the contralateral side, differences in skin barrier parameters were observed. Both desquamation index (Figure 5a) and protein content removed from D-squames (Figure 5b) were found to be significantly increased from baseline when treated with the vehicle, while a decrease was seen when using the formulation containing the extracts. Overall, variation of desquamation index and protein content were significantly different between the two sides, in favor of the BA + EN extracts treated side.

#### 2.6.2. *Betula alba* and *Empetrum nigrum* Extracts versus Niacinamide

**Figure 6:**
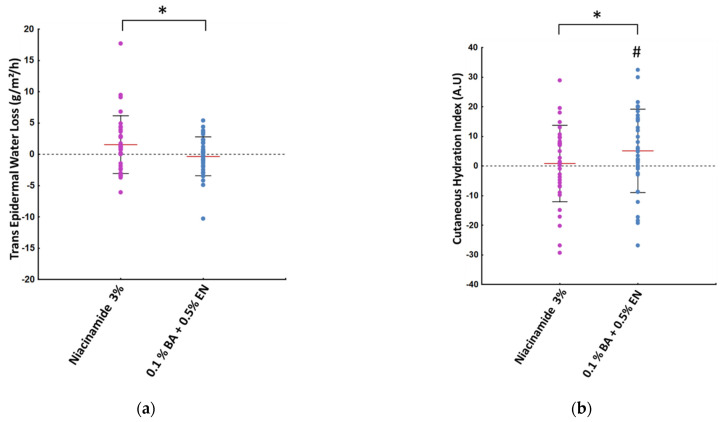
Trans-epidermal water loss (**a**) and cutaneous hydration index (**b**) after 28 days of treatment for the niacinamide (purple) and the BA + EN (dark blue) treated sides. * denotes a significant difference between the two treated sides, while # denotes a significant variation versus baseline measurements (paired Student’s *t*-test, *p* * ≤ 0.05). Red bars represent mean ± SD in black (*n* = 36).

The second study was performed on a separate cohort, which applied BA + EN extracts on one side of the face and niacinamide on the contralateral side. Significant differences in TEWL variation (Figure 6a) were seen between the niacinamide treated side and the extracts treated side, in favor of the latter. When looking at the cutaneous hydration index, substantial improvements from baseline were only observed with the extracts treated side (Figure 6b), whose variation after 28 days was significantly greater than those yielded by niacinamide.

**Figure 7:**
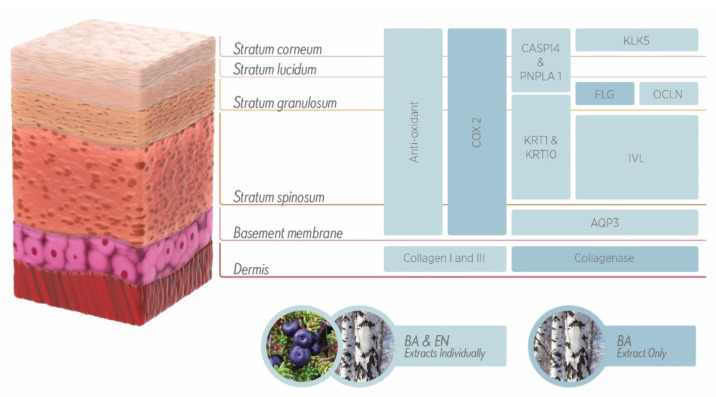
Schematic illustration of the multidirectional effects of BA and EN extracts on the skin.

An overview of the effects of BA and/or EN extracts on the skin is depicted in Figure 7.

## 3. Discussion

Niacinamide has long been considered as one of the “gold standard” ingredients in skin care due to its ability to ameliorate wrinkles, pigmentation, blemishes, and skin barrier. In this study, we explored the Scandinavian flora, for an alternative to niacinamide, to strengthen the skin barrier. Swedish plant extracts were compared for skin barrier benefits and two candidates outperformed the rest: *Betula alba* (BA) and *Empetrum nigrum* (EN). These two extracts upregulated genes as well as proteins important for skin barrier health, both when used individually and in combination. Additionally, they showed strong antioxidant and anti-inflammatory activity. These results were further supported in vivo with a restructuring effect and improved corneocyte cohesion when formulated at 0.1% for BA and 0.5% for EN extracts, versus the vehicle. Furthermore, more appreciable effects on TEWL and cutaneous hydration index were measured versus niacinamide at 3%.

Birch, or *Betula alba* has been extensively used in traditional medicine for its healing powers. Various species of birch have been shown to have antioxidant, anti-inflammatory and antibacterial effects [28]. Woelfle et al. found that treating human primary keratinocytes with triterpenes extracted from birch, induced the expression of differentiation markers keratin 10 (KRT10) and transient receptor potential 6 (TRPC6) and increased calcium ion influx [29]. The results were translated in vivo when treatment of actinic keratoses lesions resulted in normalization of skin morphology, increased expression of KRT10 and reduction of Ki67 expression.

Our study provides additional proof that BA induces keratinocyte differentiation, supported by the increased *KRT1*, *KRT10*, involucrin (*IVL*) and filaggrin (*FLG*) mRNA expression (Figure 2a). Differentiation is essential to maintain a healthy skin barrier. It involves cornification as well as desquamation, during which the cells migrate from the stratum basale to the surface of the skin where they form the stratum corneum [30]. Additionally, we show that BA has high antioxidant as well as anti-inflammatory effect. In comparison to niacinamide, BA extract induced significantly higher levels of aquaporin 3 (AQP3) and occludin (OCLN), proteins important for skin hydration and consequently skin barrier health [3,5,6].

Crowberry, or *Empetrum nigrum*, is commonly occurring in the northern hemisphere, including Europe, Canada, western Alaska and Eurasia. Among the berry crops, crowberry tops the list of families with the highest antioxidant content. Flavonoids and anthocyanins are the main group of bioactive compounds in EN, which could explain the high antioxidant activity [15]. Several studies have shown the antioxidant and anti-inflammatory effect of EN fruit [31,32,33]. Kim et al. illustrated that EN extract protected HaCat cells from UVB-induced peroxidation damage, evidenced by reduced DPPP oxide fluorescence as well as decreased protein carbonyl formation [31]. Further evidence that EN extract could suppress the release of the pro-inflammatory mediator nitric oxide (NO) was provided by Hyun et al. [32].

In our study, we corroborate that EN extract has strong antioxidant as well as anti-inflammatory activity (Figure 1 and Figure 4). Additionally, genes involved in keratinocyte differentiation are activated upon treatment with EN extract (Figure 2a). *Empetrum nigrum* extract exhibits significantly higher levels of AQP3 and OCLN proteins, compared to niacinamide, and thereby follows the same pattern as BA.

By combining BA and EN extracts in a topical formulation, we demonstrated relevant benefits for skin barrier health at a lower concentration than niacinamide, thus this dual extract is a relevant candidate for being a potential natural alternative. Nevertheless, it should be borne in mind the positive effects of niacinamide go beyond skin barrier benefits. Indeed, previous investigations have shed light on its hyperpigmentation reducing effect, fine line, and wrinkle improvement ability [34], its capacity to mitigate UV and pollution induced damage [35]. Therefore, it would be relevant to pursue investigations with this dual extract on additional skin targets. In-house investigations have revealed anti-ageing benefits (Figure S3), that deserve confirmation through clinical studies. Furthermore, formulating these two natural extracts did not pose any big challenges in terms of formulation stability, which can often arise when formulating with natural extracts.

In conclusion, we identified a natural alternative to niacinamide for improving skin barrier health, in Scandinavian plant extracts, which yield strong performance, at a lower concentration.

## 4. Materials and Methods

### 4.1. Extracts

The list of screened extracts and their composition are listed in Table 4. All extracts were used at raw concentrations, unless otherwise specified. In the instance solute concentrations are stated, it refers to the percentage of the active fraction in the extract.

The two selected extracts for further in vitro and in vivo testing following biochemical screening, were obtained the following way:

- The *Empetrum nigrum* fruit juice was obtained by cold pressing of the berries, in non-denaturing conditions, stabilized by organic vegetal glycerine.
- The *Betula alba* bark extract was obtained from an aqueous extraction process and stabilized by organic vegetal glycerine.

### 4.2. Biochemical Assays

#### 4.2.1. Kallikrein 5 Inhibition Assay

The assay measures the capacity of the extracts to inhibit the kallikrein 5 (KLK5) enzyme which can cleave the fluorogenic substrate Boc-VPR-AMC. The principle of the assay is to measure the formation of AMC, which is a highly fluorescent group (λ_exc_ = 380 nm, λ_em_ = 460 nm).

The sample extracts were dissolved and diluted at different concentrations in sodium phosphate (Fluka, 71636) buffer (0.1 M, pH 8). One microliter of sample extract at different concentration was introduced into a 96-well plate with 50 µL of KLK5 enzyme (R&D Systems, 1108-SE010) 1 ng/µL solution and incubated at room temperature for 5 min. This was followed by fluorescence pre-reading at λ_exc_ = 380 nm, λ_em_ = 460 nm, which was subtracted from each value of the final reading to avoid interference effect of colored extracts. Fifty microliter of 100 µM substrate Boc-VPR-AMC (R&D Systems, ES011) was then added to the plate and incubated 15 min at room temperature, followed by fluorescence reading at λ_exc_ = 380 nm, λ_em_ = 460 nm with spectrophotometer microplate reader Cytation 3 from Biotek.

The fluorescence intensity of a background well, sodium phosphate buffer without enzyme, was subtracted before applying the following formula to calculate percentage inhibition:

% Inhibition = [(FI Max Activity−FI sample)/FI Max Activity] × 100,

where Max Activity is the well with sodium phosphate buffer and enzyme and Sample is the well with the sample extract and enzyme. The experiment was carried out two times in triplicate. The graph was plotted with the mean response value for each concentration of extract and its standard deviation (represented with a symbol and error bar). Concentration-response curves were calculated using Prism 9 (GraphPad Software) and IC50 displayed as mean ± SD.

#### 4.2.2. Hyaluronidase Inhibition Assay

The hyaluronidase inhibition assay measures the capacity of extracts to inhibit the hyaluronidase enzyme which has ability to cleave hyaluronic acid. The principle of the assay is to precipitate the remaining hyaluronic acid with addition of albumin acidic solution and measure its turbidity at 600 nm.

The sample extracts were dissolved in enzyme diluent (20 mM sodium phosphate, 77 mM sodium chloride and 0.01% (*w*/*v*) bovine serum albumin pH 7.0 at 37 °C) at different concentrations. Twenty microliter of sample extract at different concentration were introduced into a 96-well plate with 20 µL of enzyme hyaluronidase (Sigma, St. Louis, MO, USA, H3506) 4 U/mL solution and incubated at 37 °C for 10 min. Twenty microliter of hyaluronic acid (Sigma, H5388) 0.03% (*w*/*v*), prepared in phosphate buffer (300 mM sodium phosphate, pH 5.35 at 37 °C) was then added and the plate incubated further at 37 °C for 45 min. Two hundred microliter of acidic albumin solution (24 mM sodium acetate, 79 mM acetic acid with 0.1% (*w*/*v*) bovine serum albumin, pH 3.75 at 20 °C) was added to precipitate the remaining hyaluronic acid and incubated for 20 min at room temperature followed by absorbance reading at 600 nm with spectrophotometer microplate reader Cytation 3 from Biotek. Percentage inhibition was calculated by the following formula:

% Inhibition = [(Abs Sample−Abs Max activity)/(Abs Background−Abs Max activity)] × 100,

where Background is the well with enzyme diluent and no enzyme, Max activity is the well with enzyme diluent and enzyme, and Sample is the well with sample extract and enzyme.

The experiment was carried out three times in triplicate. The graph was plotted with the mean response value for each concentration of extract and its standard deviation (represented with a symbol and error bar). Concentration-response curves were plotted using Prism 9 (GraphPad Software) and IC50 presented as mean ± SD.

#### 4.2.3. DPPH Free Scavenging Activity

The assay measures the capacity of the extracts to scavenge the radical 2,2-Diphenyl-1-picrylhydrazyl (DPPH) which at radical state is purple and absorb light at 516 nm. When DPPH is reduced by an antioxidant, it turns yellow and has no absorbance at 516 nm.

The extracts were dissolved and diluted at different concentration in methanol (Riedel-de-Haën, Seelze, Germany, 34860N). Hundred microliter of sample extract at different concentrations were introduced into a 96-well plate with 100 µL of DPPH (Sigma Aldrich, St. Louis, MI, USA, D913-2), 200 µM solution, and incubated 30 min at room temperature followed by absorbance reading at 516 nm with spectrophotometer microplate reader Cytation 3 from Biotek. Radical percentage inhibition was calculated for each concentration by the following formula:

% Radical Inhibition = [(Abs Blank−Abs Sample)]/Abs Blank,

where Blank is the well with the sample vehicle alone and Sample is the well with the sample extract.

After this, the IC50 (concentration of the extract that is required for 50% radical inhibition) could be determined. Ascorbic acid (Sigma Aldrich, 33034) was used as an assay control.

The experiment was carried out in triplicate in three independent experiments. Graph was plotted with the mean response value for each concentration of extract and its standard deviation (represented with a symbol and error bar). Concentration-response curves were calculated using Prism 9 (GraphPad Software) and IC50 presented as mean ± SD.

#### 4.2.4. COX2 Inhibition Assay

The assay measures the capacity of the extract to inhibit prostaglandin-endoperoxide synthase 2 (COX2) enzyme. The principle of the assay is to convert arachidonic acid by the enzyme COX2 to eicosanoid derivatives through two enzymatic reactions: the cyclooxygenase reaction, bis-oxygenation of arachidonic acid to prostaglandin G2 (PGG2) and the peroxidase reaction, the reduction of PGG2 to prostaglandin H2 (PGH2). The peroxidation reaction requires a second substrate Ampliflu Red which is co-oxidized during peroxidase reaction into fluorescent resorufin (λ_exc_ = 535 nm, λ_em_ = 590 nm).

The extracts were dissolved in Tris-HCl buffer (0.1 M, pH 8) (Fisher Scientific, Fair Lawn, NJ, USA, BP153-500) at different concentrations. Thirty microliter of Tris-HCl buffer (0.1 M, pH 8), 10 µL hematin (Sigma Aldrich, H3281), 10 µL of extract (1% final concentration of raw material) and 10 µL enzyme COX2 (BPS Bioscience, San Diego, CA, USA, 71111) 20 ng/µL were introduced into a 96-well plate and incubated 15 min at room temperature. Twenty microliter of co-substrate Ampliflu Red (Sigma Aldrich, 90,101) and 20 µL arachidonic acid (Sigma Aldrich, 10931) were then added and incubated 45 min at room temperature followed by fluorescence reading at λ_exc_ = 535 nm, λ_em_ = 590 nm with spectrophotometer microplate reader Cytation 3 from Biotek.

The fluorescence intensity of a background well, Tris-HCl buffer without enzyme, is subtracted before applying the following formula to calculate percentage inhibition:

% Inhibition = [(FI Max Activity−FI Sample)/FI Max Activity] × 100,

where Max Activity is the well with Tris-HCl buffer and enzyme and Sample is the well with the sample extract and enzyme. The experiment was carried out in triplicate in four individual experiments. Graph was plotted with the mean response value for each concentration of extract and its standard deviation (represented with a symbol and error bar). Concentration-response curves were calculated using Prism 9 (GraphPad Software) and IC50 presented as mean ± SD.

### 4.3. In Vitro Screening

#### 4.3.1. Cell Culture

Human primary keratinocytes all came from healthy female donors, 24–56 years of age (CellSystems GmbH, Troisdorf, Germany). They were maintained in EpiLife (Gibco, MEPI500CA) supplemented with HKGS (Gibco, S-001-5), at 37 °C in a 5% CO_2_ humidified incubator.

#### 4.3.2. Viability Assay

Viability testing was performed using CellTiter-Glo^®^ (Promega, Madison, WI, USA) according to manufacturer’s instructions. The assay measures the amount of ATP, which is directly proportional to the number of viable cells present in culture. The qualified test requirement was reached if cell viability was ≥80%. Anything below 80% was considered cytotoxic.

#### 4.3.3. Gene Expression Analysis of Barrier Genes in Keratinocytes

Quantitative polymerase chain reaction (qPCR) analysis was performed to explore the effects of *Betula alba* bark extract (BA) and *Empetrum nigrum* fruit extract (EN) on the gene expression of 10 selected skin barrier genes in human primary keratinocytes.

Keratinocytes were cultured in 48-well plates to approximately 80% confluency, followed by treatment with 0.1% solute concentration of BA or 0.5% solute concentration of EN or a combination of 0.05% BA + 0.25% EN extracts. After 24 h, RNA was extracted with RNeasy^®^ Mini Kit (Qiagen, Hilden, Germany) according to manufacturer’s instructions followed by cDNA synthesis. cDNA was synthesized with the iScript™ Advanced cDNA Synthesis Kit (Bio-Rad, Hercules CA, USA) according to manufacturer’s instructions with 15 µL of RNA in a total volume of 20 µL reaction mixture containing 4 µL 5× iScript advanced reaction mix and 1 µL iScript advanced reverse transcriptase. Quantitative PCR was performed in a total volume of 20 µL, containing 10 µL SsoAdvanced™ Universal SYBR^®^ Green Supermix (Bio-Rad), 4 µL RNase-free water, 5 µL diluted cDNA and 1 µL PrimePCR™ SYBR^®^ Green Assay (Bio-Rad) primer pair for the selected 10 barrier genes, *AQP3* (qHsaCED0046291), *OCLN* (qHsaCED0038290), *TJP1* (qHsaCID0018062), *CLDN1*(qHsaCID0006097), *FLG* (qHsaCED0036604), *CASP14* (qHsaCID0017034), *KRT1* (qHsaCID0011275), *KRT10* (qHsaCED0047830), *IVL* (qHsaCID0008540) and *PNPLA1* (qHsaCID0016270). Thermal cycling was carried out in a CFX Connect Real-Time PCR Detection System (Bio-Rad) with a program of 95 °C for 3 min, followed by 40 cycles of denaturation at 95 °C for 10 s and annealing and elongation at 60 °C for 30 s. The gene expression levels were normalized to the expression level of *GAPDH* (Bio-Rad, qHsaCED0038674) housekeeping gene. Relative gene expression changes, calculated using the 2^−∆∆CT^ method, are reported as number-fold changes compared to those in the control samples.

#### 4.3.4. Detection of Proteins Using Flow Cytometry

Human primary keratinocytes were cultured in 12-well plates in Epilife (Gibco, MEPI500CA) supplemented with 1% HKGS (Gibco, S-001-5) to a confluency of 60–70%. Cells were treated with 0.025% solute concentration of BA, 0.125% solute concentration of EN or 0.1% niacinamide for 48 h. After 48 h treatment, cells were washed and fixed (Invitrogen, 88-8823-88) followed by staining for AQP3 (Nordic Biosite, bs-1253R-A488) and OCLN (SantaCruz, c-271842 AF647) protein expression both on surface markers and intracellularly for 1 h in fridge and room temperature, respectively. After staining, cells were strained, and protein expression analysed with flow cytometry (ACEA NovoCyte 2000R). The median value was used for analysis and the unstained sample’s median from respective treatment was subtracted from stained samples of the same treatment to normalize for autofluorescence. Statistics were performed on subtracted data.

### 4.4. Extract Identification

#### UHPLC/Q-ToF Analysis

*Betula alba* was diluted 10 times in 95:5 H_2_O:ACN and filtered through a 0.2 µm syringe filter. *Empetrum nigrum* was used without dilution and was filtered through a 0.2 µm syringe filter prior to analysis. Both solutions were analysed using an Agilent UHPLC/Q-ToF (1290 Infinity UHPLC and 6520 Q-ToF) with an electrospray ionization source (ESI).

The separation was performed on a Zorbax extend C_18_ 2.1 × 150 mm, 1.8 µm column kept at 35 °C at a flow rate of 0.25 mL/min. The injection volume was 20 µL. The mobile phase consisted of H_2_O + 0.1% FA (A) and ACN + 0.1% FA (B), using the following gradient: 98% A for the first 10 min which was decreased to 40% after 70 min and finally to 2% after 90 min. Negative ESI was used for BA and +ESI for EN with the following settings: gas temperature 350 °C, drying gas 8 L/min, nebulizer 40 psig and Vcap 3500 V.

### 4.5. In Vivo Testing

#### 4.5.1. Study 1: Vehicle Controlled Study

##### Panel

Thirty-five female volunteers aged 18–65 (mean age = 47, SD = 15) were recruited from Lyon and nearby area in France. All procedures involved in the study were explained in detail and informed consent was obtained from all volunteers. Enrolment was definite when volunteers met all inclusion and exclusion criteria from this double-blind, randomized and within-subject study. Main study inclusion criterion was self-perceived dry to very dry skin. Exclusion criteria were as follows: pregnant or nursing woman or woman planning to get pregnant during the study, start/stop/change in hormonal treatment (including contraceptive pill) <1.5 months, cutaneous pathology on the study zone (face), use of topical or systemic treatment during the previous weeks liable to interfere with the assessment of the cutaneous efficacy of the study products, known allergy to certain cosmetic or pharmaceutical products, subject having done injections on face and/or a lifting procedure, excessive exposure to sun or UV rays in the previous month and volunteers having undergone surgery under general anaesthesia within the previous month. Prior to baseline measurements, all volunteers went through a wash out phase of two weeks, where volunteers were instructed to apply a simple moisturizer instead of their regular products. Following a period of 15 min acclimatization under controlled temperature and humidity (22 °C ± 2 °C, relative humidity between 35% and 55%), volunteers went through a set of measurements at baseline (day 0) and 28 days. The volunteers were instructed to apply the formulation containing 0.1% BA + 0.5% EN extracts (solute concentration) on one half of the face, and the corresponding vehicle formulation on the contralateral side, twice daily, for a duration of 28 days. The composition of the vehicle formulation is shown in Table 5. Allocation of products to the left or right side was randomized. The study was conducted under good clinical practice (GCP) and in conformance with the most recent recommendations of the World Medical Association [36].

##### Measurements

For each volunteer, standard D-Squame^®^ discs with a diameter of 2.2 cm and an area of 3.8 cm^2^ were placed on the left and right temple areas, under 225 g/cm^2^ of pressure, using a pressure device (CuDerm Corporation). The D-Squame^®^ was then removed in one fluid and rapid movement. The procedure was repeated with a second D-squame^®^ disc.

Two different types of analysis were then performed using the D-Squame discs:

- Restructuring effect using the SquameScan^®^ 850A (Heiland Electronic). It measures the stratum corneum protein content on D-Squame^®^ tape strips, which is achieved by measuring the optical absorption of the strip at 850 nm (infrared light). The value is displayed in %.
- Corneocyte cohesion using Skin Image Analyser^®^ (S.I.A^®^) with the QuantiSquam^®^ software. The surface of stripping is lightened in a standardized way (35°) and observed with a digital camera linked to a computer. The digitized image obtained is analysed in grey levels to determine the desquamation index (ratio between the occupied surface and the thickness of the cellular layer).

#### 4.5.2. Study 2: Comparison of BA + EN Extracts Versus Niacinamide

##### Panel & Products

As for the second study, which focused on the performance of the dual extract versus niacinamide, a cohort of 36 female volunteers aged 18–65 (mean age = 49, SD = 13) was recruited. Inclusion and exclusion criteria were the same as outlined in 4.5.1. Products applied were the vehicle containing niacinamide at 3% on half of the face, and the vehicle containing 0.1% BA + 0.5% EN extracts (solute concentration) on the contralateral side. Inclusion levels of niacinamide were chosen according to levels reported in the literature for skin barrier benefits [9,37].

##### Measurements

Trans-epidermal water loss (TEWL) was measured using an AquaFlux AF103 (Biox Systems). The AquaFlux^®^ chamber consists of a small cylinder, closed in its upper end with a condenser to maintain the temperature (−7.6 °C) below the freezing temperature of water by means of a Peltier cooler. This system allows controlling the humidity in the chamber independently of ambient conditions (variations in temperature and hygrometry), by converting water vapor into ice, thus maintaining low humidity.

Cutaneous hydration index was measured using the MoistureMap MM100^®^ (CK Electronic GmbH). The probe has a size of 4.3 × 3 cm and a measurement surface of 18 × 12.8 mm with more than 90,000 micro-capacitors located every 50 μm. This configuration allows a measure of the penetration of the electromagnetic field by capacitance imaging, and thus visualization of the hydration topography. Capacitance values are transformed into pixels, on a scale of 256 gray levels: darker pixels (=important reflected signal) represent the more hydrated zones. On the contrary, a dryer zone gives a lower signal. All measurements were performed on the temple area.

### 4.6. Statistics

Statistics for all in vitro studies were calculated in Prism 9 (GraphPad Software). Statistically significant differences between samples were evaluated by a repeated measures ANOVA with Tukey’s multiple comparison test as post-test or paired Student’s *t*-test and were assumed significant at *p* * ≤ 0.05, *p* ** ≤ 0.01 and *p* *** ≤ 0.001. For gene expression, statistics was performed on ΔCt values while for protein expression normalized values were used.

For in vivo studies, the normality of data was assessed using the Shapiro–Wilk test, with a threshold of α = 0.01. Accordingly, paired Student’s *t*-test (if normality assumed) or Wilcoxon signed-rank test (normality assumption rejected) were used for baseline comparisons and between-treatment comparisons, with a significance level set at α = 0.05 throughout the analysis. All calculations were performed using STATISTICA V14 (Tibco).

## Figures and Tables

**Table 1:** List of six Scandinavian plants, and the resulting plant extracts.

Binomial Name	Common Name	Plant Part Used	Abbreviation
*Betula alba*	Birch	Bark	BA
*Empetrum nigrum*	Crowberry	Fruit	EN
*Hippophae rhamnoides*	Sea-buckthorn	Seed	HR
*Rhodiola rosea*	Roseroot	Stem cell	RR
*Trifolium pratense*	Red clover	Flower	TP
*Vaccinium vitis-idaea*	Lingonberry	Stem cell	VVI

**Table 2:** List of genes screened by quantitative PCR.

Gene	Name	Protein Function
*AQP3*	Aquaporin 3	A membrane transporter of water and glycerol expressed in the basal layer of epidermis in normal skin. It is important for maintaining water content and elasticity of the skin.
*OCLN*	Occludin	Together with CLDN1 and TJP1, the main component of the tight junctions.
*TJP1*	Tight junction protein 1	Together with CLDN1 and OCLN, the main component of the tight junctions.
*CLDN1*	Claudin 1	Together with TJP1 and OCLN, the main component of the tight junctions.
*FLG*	Filaggrin	Essential for the regulation of epidermal homeostasis. Filaggrin monomers can incorporate into the lipid envelope, which is responsible for the skin barrier function.
*CASP14*	Caspase 14	Required for the degradation of FLG into natural moisturizing factors (NMFs) in the skin. Blocking the FLG processing done by Caspase-14, results in defects in water retention.
*KRT1*	Keratin 1	Differentiation of keratinocytes from the basal to the spinous layer is characterized by a shift to KRT1 and KRT10. The primary function of the keratin intermediate filament cytoskeleton is to provide cells with structural resilience against mechanical trauma.
*KRT10*	Keratin 10	Differentiation of keratinocytes from the basal to the spinous layer is characterized by a shift to KRT1 and KRT10. The primary function of the keratin intermediate filament cytoskeleton is to provide cells with structural resilience against mechanical trauma.
*IVL*	Involucrine	A cornified envelope protein. Together with keratins, it is responsible for the mechanical stability of the corneocytes. Involucrine binds covalently to ceramides, forming a backbone for the subsequent attachment of free ceramides.
*PNPLA1*	Patatin-like phospholipase domain-containing 1	An enzyme expressed in differentiated keratinocytes, which plays a crucial role in the biosynthesis of ω-O-acylceramide, a lipid component essential for skin barrier integrity.

**Table 3:** List of compounds tentatively identified in BA and EN. UHPLC/ESI-Q-ToF in negative mode was used for BA and positive mode for EN.

	Empirical Formula	Calculated *m/z*	Retention Time (min)
**BA**		**−ESI**	
Catechin 7-xyloside	C_20_H_22_O_10_	421.1140	18.3
Catechin	C_15_H_14_O_6_	289.0718	18.8
Apiosylepirhododendrin	C_21_H_32_O_11_	459.1872	23.8–24.6
Rhododendrin	C_16_H_24_O_7_	327.1449	24.1
Platyphylloside	C_25_H_32_O_9_	475.1974	27.4–32.6
5-Hydroxy-1,7-bis-(4-hydroxyphenyl)-3-heptanone 5-O-β-D-apiofuranosyl-β-D-glucopyranoside	C_30_H_40_O_13_	607.2396	32.0
1,7-Bis-(4-hydroxyphenyl)-3-heptanol-3-O-[2,6-bis-O-(β-D-apiofuranosyl)-β-D-glucopyranoside	C_35_H_50_O_16_	725.3026	36.2
Aceroside VIII	C_30_H_42_O_12_	593.2603	36.8–37.4
5-Hydroxy-3-platyphyllone	C_19_H_22_O_4_	313.1445	38.0
Aceroside VIII	C_30_H_42_O_12_	593.2603	36.8–37.4
Centrolobol	C_19_H_24_O_3_	299.1653	46.5
Acerogenin E	C_19_H_20_O_3_	295.1340	47.5
**EN**		**+ESI**	
Cyanidin 3-galactoside	C_21_H_21_O_11_^+^	449.1089	19.7
Petunidin 3-galactoside	C_22_H_23_O_12_^+^	479.1195	20.5
Peonidin 3-galactoside	C_22_H_23_O_11_^+^	463.1246	21.8
Malvidin 3-galactoside	C_23_H_25_O_12_^+^	493.1351	22.3
Delphinidin 3-arabinoside	C_20_H_19_O_11_^+^	435.0933	30.0

**Table 4:** List of the six extracts screened and their composition.

Plant	INCI Name
*Betula alba*	Glycerin, water, *Betula alba* bark extract
*Empetrum nigrum*	Glycerin, *Empetrum nigrum* fruit juice
*Hippophae rhamnoides*	Maltodextrin, *Hippophae rhamnoides* kernel extract
*Rhodiola rosea*	Glycerin, water, *Rhodiola rosea* callus extract
*Trifolium pratense*	Isopentyldiol, *Trifolium pratense* flower extract
*Vaccinium vitis-idaea*	Water, glycerin, *Vaccinium vitis-idaea* fruit extract, xanthan gum, sodium benzoate, citric acid, gluconolactone, calcium gluconate

**Table 5:** Ingredient list of vehicle formulation used during the clinical studies. Ingredients are listed in decreasing order from top to bottom.

Vehicle Formulation Ingredient List
Water Caprylic/capric triglyceride
Butylene glycol
C12-15 alkyl benzoate
Cyclopentasiloxane
PPG-3 benzyl ether myristate
Polyacrylate crosspolymer-6
Glyceryl caprylate
Ethylhexylglycerin
Cetyl alcohol
Glyceryl stearate
PEG-100 stearate
Stearyl alcohol
Xanthan gum
Glycerin
Disodium EDTA
Caprylhydroxamic acid
Sodium hydroxide
Pentaerythrityl tetra-di-t-butyl hydroxyhydrocinnamate

## Data Availability

The data presented in this study are available on request from the corresponding authors.

## Supplementary Materials

The supporting information can be downloaded at: https://www.mdpi.com/article/10.3390/ijms232012507/s1.
