# Supplementary material for: Betula alba Bark Extract and Empetrum nigrum Fruit Juice, a Natural Alternative to Niacinamide for Skin Barrier Benefits
Source: Int J Mol Sci. 2022 Oct 19;23(20):12507. doi: 10.3390/ijms232012507 (PMC9604162; doi:10.3390/ijms232012507)
Supplement: Supplementary file 1 [file ijms-23-12507-s001.zip › ijms-1916815-supplementary.pdf]

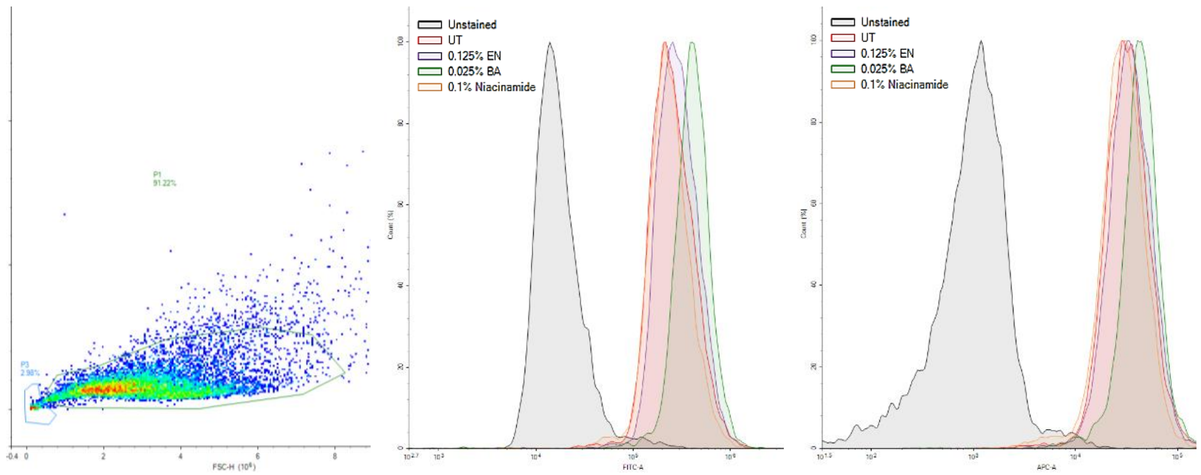

(a) **(b)** **(c)**

Figure S1: Representative density plot (a) and histograms of protein expression of AQP3 (b) and OCLN (c) in keratinocytes after 48h treatment with untreated (red), 0.025% BA (green) or 0.125% EN (blue). As a reference, 0.1% niacinamide (orange) was used. The grey histograms represent unstained sample.

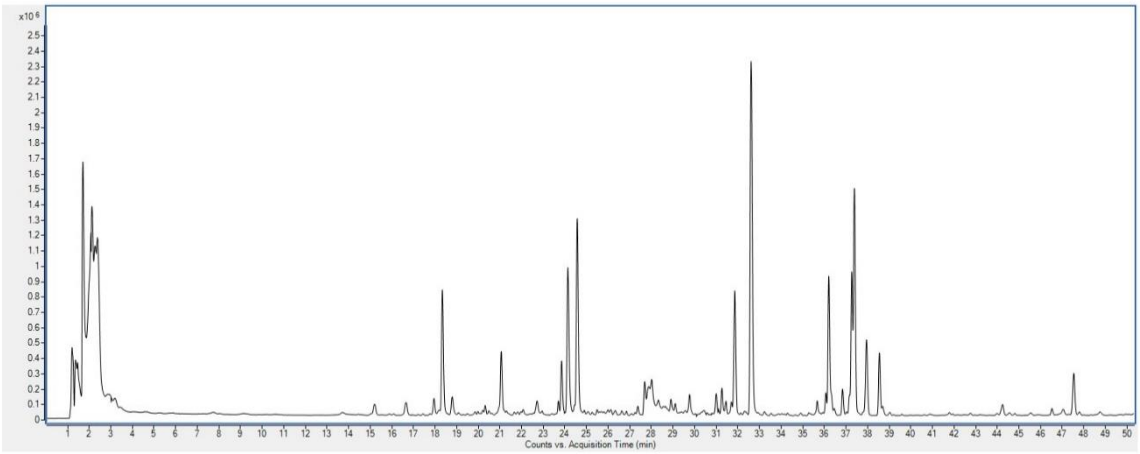

(a)

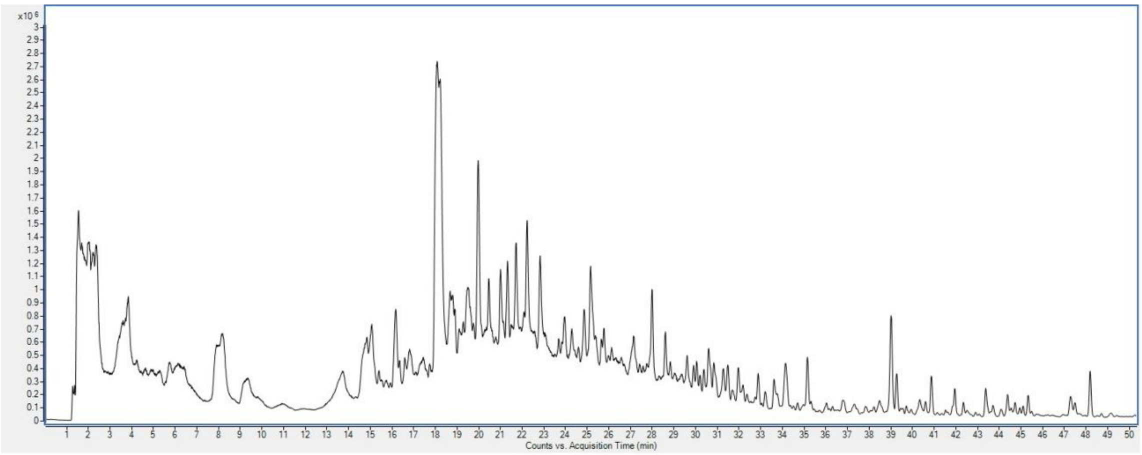

(b)

Figure S2: UHPLC/ESI-Q-ToF chromatograms of BA (a) and EN (b) extracts.

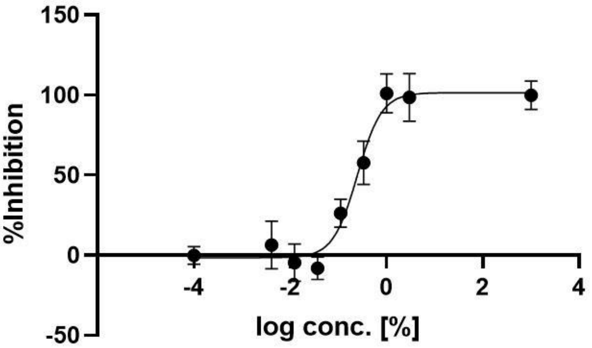

(a)

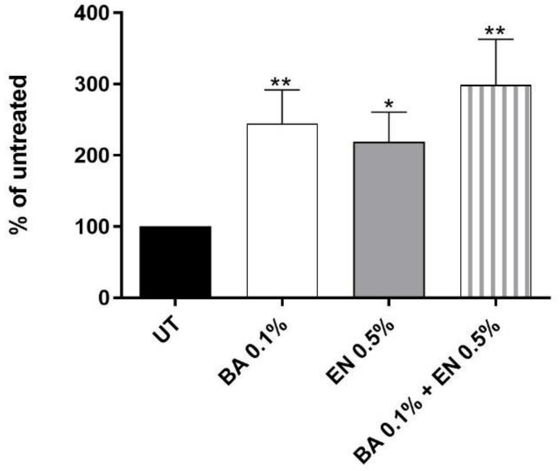

(b)

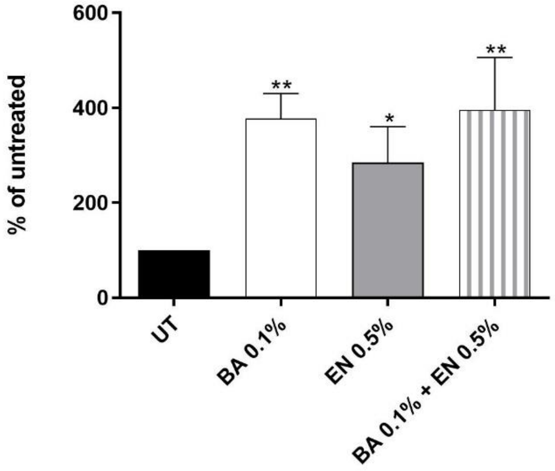

(c)

Figure S3: IC50 curve for BA extract in collagenase assay (a) where (n=3). Collagen I (b) and collagen III (c) protein levels after 48h treatment of human dermal fibroblasts with 0.01% solute concentration of BA, 0.5% solute concentration of EN or 0.1% BA + 0.5% EN (solute concentration), relative to untreated control. Values are displayed +/-SEM, n=3 donors, where each experiment was performed once. A one-way ANOVA, repeated measures test with Dunnett's post-test was performed for statistical analysis where * $p < 0.05$ and ** $p < 0.01$.
